# A Scalable, Modular Degasser for Passive In-Line Removal of Bubbles from Biomicrofluidic Devices

**DOI:** 10.3390/mi14020435

**Published:** 2023-02-11

**Authors:** Hannah B. Musgrove, Amirus Saleheen, Jonathan M. Zatorski, Abhinav Arneja, Chance John Luckey, Rebecca R. Pompano

**Affiliations:** 1 Department of Chemistry, University of Virginia, Charlottesville, VA 22904, USA; 2 Department of Pathology, University of Virginia School of Medicine, Charlottesville, VA 22904, USA

**Keywords:** bubble trap, CNC machined, 3D printing, bubble removal, microfluidics, FDM printing

## Abstract

Bubbles are a common cause of microfluidic malfunction, as they can perturb the fluid flow within the micro-sized features of a device. Since gas bubbles form easily within warm cell culture reagents, degassing is often necessary for biomicrofluidic systems. However, fabrication of a microscale degasser that can be used modularly with pre-existing chips may be cumbersome or challenging, especially for labs not equipped for traditional microfabrication, and current commercial options can be expensive. Here, we address the need for an affordable, accessible bubble trap that can be used in-line for continuous perfusion of organs-on-chip and other microfluidic cultures. We converted a previously described, manually fabricated PDMS degasser to allow scaled up, reproducible manufacturing by commercial machining or fused deposition modeling (FDM) 3D printing. After optimization, the machined and 3D printed degassers were found to be stable for >2 weeks under constant perfusion, without leaks. With a ~140 µL chamber volume, trapping capacity was extrapolated to allow for ~5–20 weeks of degassing depending on the rate of bubble formation. The degassers were biocompatible for use with cell culture, and they successfully prevented bubbles from reaching a downstream microfluidic device. Both degasser materials showed little to no leaching. The machined degasser did not absorb reagents, while the FDM printed degasser absorbed a small amount, and both maintained fluidic integrity from 1 µL/min to >1 mL/min of pressure-driven flow. Thus, these degassers can be fabricated in bulk and allow for long-term, efficient bubble removal in a simple microfluidic perfusion set-up.

## 1. Introduction

Perfusion or exchange of nutrient rich media is essential for long term cell culture in microfluidic devices. In addition to nutrients, however, cell media also usually contains dissolved gases. These gasses can emerge out of solution as bubbles, which in turn can interrupt the fluid flow. In a microscale culture setting, bubbles that stagnate in the chip cause major challenges, including obstructing nutrient delivery and waste removal, altering the gas environment, and damaging soft materials. Bubbles are difficult to prevent, as any increases in temperature, decreases in pressure [1], or roughness in channel or junctions can encourage bubble generation [2].

Many methods for reducing and removing bubbles from perfused media have been developed to address this issue. A standard strategy is to pre-equilibrate the media in conditions matching the culture (e.g., 37 °C and 5% CO_2_); this step reduces the quantity of bubbles that form in the flow path, but it cannot completely prevent them. Once formed, bubbles must be removed from the fluid either by further degassing in situ or by “trapping” bubbles to sequester them away from the culture [3,4,5]. Both active and passive microfluidics have been developed for inline bubble removal. Active systems are effective but can be complex in their setup and fabrication, which drives up the cost and may require inconvenient integration of external vacuum or electronic controllers into microfluidic culture systems. Active approaches include ultrasonic micro-degassing [6], use of hydrophobic plates [7], and pressure-based vacuum systems [8]. Passive removal systems, on the other hand, often offer simpler chip integration without external utility requirements [3]. Passive strategies include placement of gas-permeable membranes over compressed channel entries [9,10], designs that impose pressure gradients [10,11], and structures that utilize bubble buoyancy [12,13].

Even with all of these choices, selecting a user-friendly system for perfusion of on-chip cultures remains a challenge. This can be especially true for degassing large volumes (mLs or 100s of mLs) of perfused media over many days, as a higher degassing capacity is required compared to degassing of precious solutions in-situ, for instance [5,10]. Furthermore, the fabrication materials must be compatible with aqueous cell culture reagents, sterilization (e.g., with ethanol), and physiological temperatures. The degasser-to-tubing connections must be easily adaptable to maintain modularity with various chips in a leak-free manner. Just as importantly, the degasser should be easy to obtain and implement by laboratories which do not have an interest in hand-building a custom bubble trap, nor in setting up vacuum or electronics in their incubators. Thus, perfusion-based microscale cell cultures would benefit from a degassing module that can be mass produced inexpensively and reproducibly, integrates modularly into the flow path, has a passive design, and does not contribute to cytotoxicity in the culture. Progress was made in 2010 in an elegantly simple design presented by Zheng et al., in which a PDMS module containing a large reservoir was placed in the path of the media flow [12]. The inlet to the reservoir was placed higher than the outlet, passively restricting bubbles from entering the chip due to buoyancy. Since then, this device has been successfully implemented in PDMS by us and others [14,15,16,17]. The next step to increase its utility was to move away from manual assembly and adapt the design for more scalable manufacturing methods and materials.

**Figure 1:**
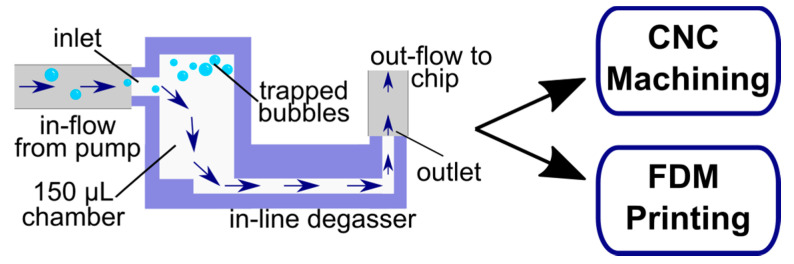
Schematic showing the design and principle of the passive, modular degasser, which was adapted for fabrication by CNC machining or FDM 3D printing.

Both 3D printing and commercial machining offer the possibility of moderate to high-throughput fabrication and multiple reuses of devices to decrease device cost per use [18,19]. Therefore, inspired by the work of Zheng et al. [12], here we adapted a buoyancy-based degassing module, also known as “bubble trap”, for implementation with these two fabrication strategies (Figure 1). For moderate-scale, in-house production, we adapted the degasser design for fused deposition modeling (FDM) 3D printing in polylactic acid (PLA). For large-scale production, we implemented it using commercial machining in polystyrene. All degassers were designed to be connected just upstream of the chip as a modular unit. We tested all bubble traps for cytocompatibility with environmentally sensitive primary cell cultures, bubble removal efficacy, and fluidic integrity to create an easily adaptable, scalable device.

## 2. Materials and Methods

### 2.1. Chamber Design Adaptation

Based on the bubble trap design adapted from Zheng et al. [12], we used a ~150 µL cylindrical chamber with an inlet placed near the top of the chamber. The chamber was 5 mm in diameter with a height of 7 mm–7.7 mm depending on manufacturing; this chamber can be increased in size to accommodate larger volumes of media and bubbles. The outlet channel was 1 × 1 × 13 mm^3^ (w, h, l) and was oriented below the chamber. The dimensions were kept constant for all fabrication methods except where noted. All features were designed with AutoDesk Fusion 360 2020, and files were exported to an STL format for use with MiiCraft 3D printing software. Outer features and tubing connections were edited as needed for optimized fabrication and connections to either a Fusion Series syringe pump (Chemyx Inc., Stafford, TX, USA) or an Ismatec™ IPC High Precision Multichannel peristaltic pump (Cole-Parmer, Vernon Hills, IL, USA).

### 2.2. Device Fabrication by Micromachining

Polystyrene devices were computer numerical control (CNC) machined by Aline Inc. (Signal Hill, CA, USA) in two parts, and sealed with pressure sealed adhesive (PSA). Threaded ports were added to integrate Zero Dead Volume Barbs (polypropylene, coupling fitting, 10-32 port, 1/16” barb, NResearch, West Caldwell, NJ, USA), for tubing connections to and from the bubble trap. Flexible high-temperature silicone rubber tubing (Soft, Durometer 50A, 1/16” ID, 1/8” OD, Semi-Clear White, McMaster-Carr, Elmhurst, IL, USA) was used to bridge the barb fitting to peristaltic tubing (Tygon S3 Tubing, 0.078” OD, 0.008” ID, Cole-Parmer, USA).

### 2.3. Device Fabrication by Fused Deposition 3D Printing

For fused deposition manufactured (FDM) devices, polylactic acid (PLA) filament (natural clear, 0.5 kg spool, Monoprice, Rancho Cucamonga, CA, USA) was used in an Ender-3 v2 Neo printer (Firmware v1.1.4, Creality, Shenzhen, China). Design files were prepared for printing using Ultimaker Cura v. 5.1.1 and were optimized for material shrinkage by scaling the design file up by 3%. The temperature of the extruder was set to 220 °C with a 60 °C base. The layer height was set to 0.12 mm for each print layer, along with a 50% gyroid infill, with 7 perimeter shells, 7 solid bottom, and 7 top layers. Print speed was set to 20 mm/s for the infill and 10 mm/s for the walls. Once removed from the baseplate, extra stringing and rough edges were removed via sanding using P1000 grit sandpaper.

To ensure snug connections between the initial tubing and chip, the printed barbed ports were wrapped in polytetrafluoroethylene (PTFE) piping tape (Grainger Inc., Richmond, VA, USA) and connected to either 1/8” PTFE bridge tubing (Ramé-Hart, Succasunna, NJ, USA) or 1/16” silicone rubber tubing (McMaster-Carr, USA). Connected bridge tubing was sized down by either direct connection to the primary tubing or by using miniature barbed polypropylene fittings (Cole-Parmer, USA). For use with peristaltic pumps, connections were using the same silicone rubber and Tygon S3 tubing as in Section 2.2. For use with syringe pumps, the polypropylene adaptors were used to connect the 1/8” PTFE bridge tubing to a black TT-30 PTFE tubing (0.012”/0.021” ID/OD, Weico Wire, Brentwood, NY, USA).

### 2.4. Sterilization and Collection of Effluent

All bubble traps and microfluidic tubing were first sanitized by thoroughly rinsing all internal and external surfaces with a 70% ethanol solution, followed by two rinses with 1x sterile Dulbecco’s phosphate buffer solution (PBS, no calcium, no magnesium, Gibco, Paisley, Scotland, UK) for all internal features. Pieces were dried in a sterile biosafety cabinet with compressed nitrogen. All bubble trap exteriors were additionally sanitized with a low dose of UV light for 20 min.

To collect effluent for cytocompatibility tests, 3 mm plastic syringes (BD, Franklin Lakes, NJ, USA) were connected to the inlet of the bubble traps using black TT-22 PTFE tubing (0.028”/0.038” ID/OD, Weico Wire, USA). Bubble traps were placed in a cell culture incubator at 37 °C. Syringes were filled with 2 mL of CTS^TM^ AIM-V media with 50 μg/mL streptomycin sulfate and 10 μg/mL gentramicin sulfate (Gibco, UK). A Fusion 200 syringe pump (Chemyx, USA) was used to deliver 1.7 mL of media overnight at a rate of 1 µL/min (>24 h), and the effluent was collected and stored at 0–2 °C until use.

### 2.5. Cell Sourcing and Determination of Biocompatibility

Human naïve CD4+ T cells used in this work were sourced and prepared as described previously [14]. Briefly, T cells were purified from TRIMA collars from healthy donors (collars purchased from INOVA Laboratories, Sterling, VA, USA) and were isolated using human CD4+ T cell RosetteSep™ kit (STEMCELL Technologies, Vancouver, BC, Canada) and Ficoll-Paque (Cytiva Inc., Marlborough, MA, USA) density centrifugation, and enriched by immuno-magnetic negative selection with the EasySep™ Naïve CD4+ T cell isolation kit (STEMCELL Technologies).

The conditioned media (effluent) from Section 2.4 was used to culture primary human naïve CD4+ T cells at 1 × 10^6^ cells/mL in a tissue culture treated 96-well plate for 24 h. Cell viability was assessed by staining cells with 10 μM Calcein-AM and 1 μM DAPI in PBS for 30 min at 37 °C, rinsing with 5× volume PBS, and imaging with a Zeiss AxioZoom microscope. ImageJ was used to identify and count cells, using the Count Particles function with a circularity of 0.5–1 and a size 12.5–500 µm^2^. Percent viability was defined as Calcein positive/(Calcein positive + DAPI positive).

### 2.6. Determination of Bubble Trapping Efficacy and Fluidic Integrity

AIM-V media was prepared by bringing it to room temperature, then equilibrated at 37 °C overnight (for machined degassers) or for ~1–2 h (for 3D printed degassers). All degassers were connected to an Ismatec™ Multichannel peristaltic pump (Cole-Parmer, USA) with Tygon S3^TM^ tubing (0.078/0.008 in OD/ID) and placed in an incubator at 37 °C for 2 weeks, during which the prepared media was pumped through the devices at a rate of 1 µL/min. Afterwards, the devices were evaluated quantitatively for effectiveness (i.e., bubble content) and periodically evaluated qualitatively for leaking or other material deformities.

### 2.7. Determination of Absorption

Rhodamine dye stock solution (Rhodamine WT, 20% in water, Acros Organics, Morris Plains, NJ, USA) was diluted 1:10 vol/vol in deionized (DI) water. Machined and FDM degassers were first rinsed with water, then filled with the diluted rhodamine. A PDMS chip was also rinsed and filled with the solution for comparison. The rhodamine-filled degassers and chips were incubated overnight (20 h). Once removed, the bubble traps were manually flushed with DI water for 20 s, followed by a brief rinse with 70% ethanol, and again with running DI water for 20 s. After rinsing, the devices were evaluated for any dye absorption via imaging. All chips were imaged on a Zeiss AxioZoom Microscope equipped with CL9000 LED brightfield source, HXP 200C metal halide lamp, PlanNeoFluor Z 1x objective (0.25 NA, FWD 56 mm), a FL Filter Set HE 43 Rhodamine filter cube (ex: 550/25 nm, em: 605/70 nm), and an Axio 506 mono camera (Carl Zeiss Microscopy, Jena, Germany). Images were analyzed using ImageJ.

### 2.8. Determination of Leaching

AimV media was allowed to perfuse through both machined and FDM printed degassers overnight (20 h at 37 °C) as described in Section 2.4. Media was then collected from the bubble traps and UV-Vis absorbance was evaluated from 200 nm to 700 nm with a NanoDrop ND 1000 Spectrophotometer (ThermoFisher, Waltham, MA, USA). Absorbance at 280 nm was quantified. Non-perfused media was used as a control.

### 2.9. Determination of Mechanical Integrity under Varied Pressure Drops

To assess the performance of the degassers as a function of applied fluid pressure, a Push-Pull, Vacuum Pressure-Based Controller (Fluigent, Le Kremlin-Bicêtre, France) was connected to a water-filled reservoir, from which peristaltic tubing was connected to a bubble trap as in Section 2.3. The reservoir was slowly pressurized and chips were evaluated for any signs of leaks throughout the process. The flow rate was estimated by timing the collection of 2 mL of water from the outlet of the degasser.

### 2.10. In-Line Device Application

To test the efficacy of bubble removal downstream, the degassers were tested in series with a previously reported microfluidic culture chip fabricated in PDMS and glass [14]. Machined and FDM printed degassers were placed upstream of one culture chip each, and an Ismatec™ peristaltic pump was used to perfuse AIM-V media overnight (20 h) at a rate of 1 µL/min. Culture chips without degassers were used as a control. The culture chips were then imaged on the Zeiss AxioZoom Microscope (Carl Zeiss Microscopy). Images were analyzed for the presence of bubbles with Zeiss Zen 3.3. Blue software.

## 3. Results and Discussion

Here we report the successful adaptation and optimization of a passive bubble trap design for two automated manufacturing methods, CNC manufacturing and 3D printing. In both cases, the design included a reservoir chamber, an inlet near the top of that chamber, and a channel at the bottom of the chamber to separate bubbles from the line of flow (Figure 1). The manufactured devices were fitted with barbed connections, allowing them to be inserted as a module between a pump and a downstream culture device. Due to this modularity, the degassers could be reused for multiple experiments by disconnecting the degasser from the pump and chip and cleaning as needed. Further design development and validation of the two fabrication methods are described below along with a demonstration of this degasser with our previously described microfluidic chamber [14].

### 3.1. Micromachined Degassers

**Figure 2:**
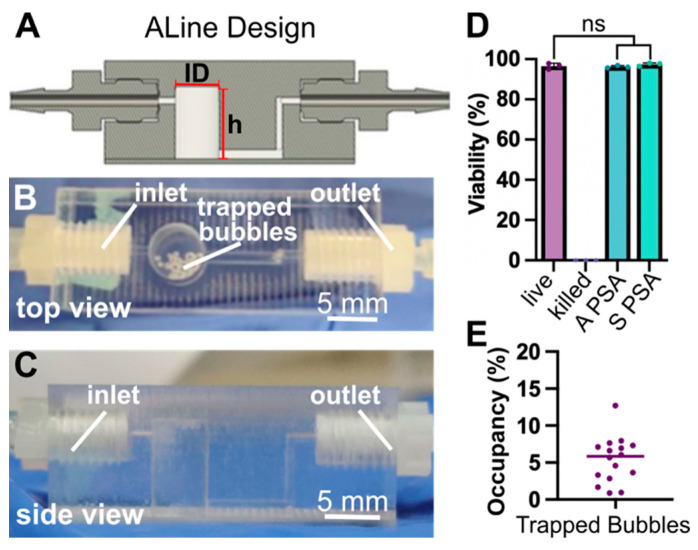
Machined degassers efficiently trapped bubbles for prolonged experiments and maintained biocompatibility with human T cell cultures. (**A**) A schematic of the machined degasser. ID = 5 mm, h = 7.726 mm. (**B**) Top and (**C**) side view of the machined degasser in use, with bubbles visible in the traps. (**D**) Quantification of viability of human T cells cultured in conditioned media that was collected as effluent from degassers, compared to live and ethanol-killed plate controls. The degassers were fabricated with either acrylic pressure sealant adhesive (A PSA) or silicone pressure sealant adhesive (S PSA). Viability is defined as percent of cells positive for calcein. One-way ANOVA, ns *p* > 0.4. (**E**) The percent of the degasser chamber occupied by bubbles after 14 days of media perfusion at 37 °C. All data points were less than 15% of total available capacity, with a median of 5.8% capacity. Each data point represents one bubble trap, and the line represents the median.

To adopt a scalable approach for fabrication, polystyrene degassers were produced by ALine Inc. (Signal Hill, CA, USA), by CNC machining (Figure 2A–C). To facilitate machining, the device was manufactured in 2 layers, which were fused with pressure-sensitive adhesive (PSA). Threading was included to fit zero-dead-volume barbs at the inlet and outlet. The chamber maintained a capacity of 151 µL. To accommodate the inlet tubing from the peristaltic pump and the outlet tubing to the chip, a set of bridge tubing was used to ensure a snug fit (see Methods). These fittings could be adapted as needed to accommodate a variety of tubing by changing the barb size or by using adaptor fittings.

Two different types of PSA were tested for biocompatibility in the device, Acrylic (A PSA) and Silicone (S PSA). To test biocompatibility, media was perfused through the bubble trap at a rate matched to our standard cell culture experiments. The effluent (conditioned media) was collected, and these media were used to culture human CD4+ T cells overnight. We observed that media contact with the machined degassers did not alter T cell viability compared to live controls, irrespective of the type of PSA used (Figure 2D and Figure S1). Therefore, both types of traps were deemed suitable for use with biomicrofluidics, and the A-PSA and S-PSA degassers were pooled for subsequent studies.

Bubble trap capacity in this design is limited by the volume of the reservoir, so we tested its suitability for multi-day and multi-week culture under a representative set of culture conditions based on our prior work [14]. Media was placed in an incubator at 37 °C and then perfused slowly through the trap inside the cell culture incubator. An average of 4–6% of the degasser reservoir capacity was occupied by bubbles at the end of the 14-day perfusion period (Figure 2E). Assuming a constant rate of bubble formation under the same conditions, it would take upwards of 20 weeks to fill the capacity of the chamber to 50%, thus providing the potential for long-term cell culture experiments. We arbitrarily selected 50% as the maximum allowable percent occupancy, as above a certain point the bubbles may be at risk of flowing out of the chamber or causing fluctuations in flow rate.

In summary, the micromachined bubble traps were effective in bubble removal, biocompatible, and provided easy connections using commercially available barbs. The polystyrene offered good transparency for visualization of bubbles. Micromachining offers a highly consistent, high-throughput fabrication option, with affordability increasing as the amount of bubble traps increases. This is a good option if many bubble traps (on the scale of ~100 or greater) are required, though potentially impractical for smaller batches.

### 3.2. Fused Deposition 3D Printed Degassers

**Figure 3:**
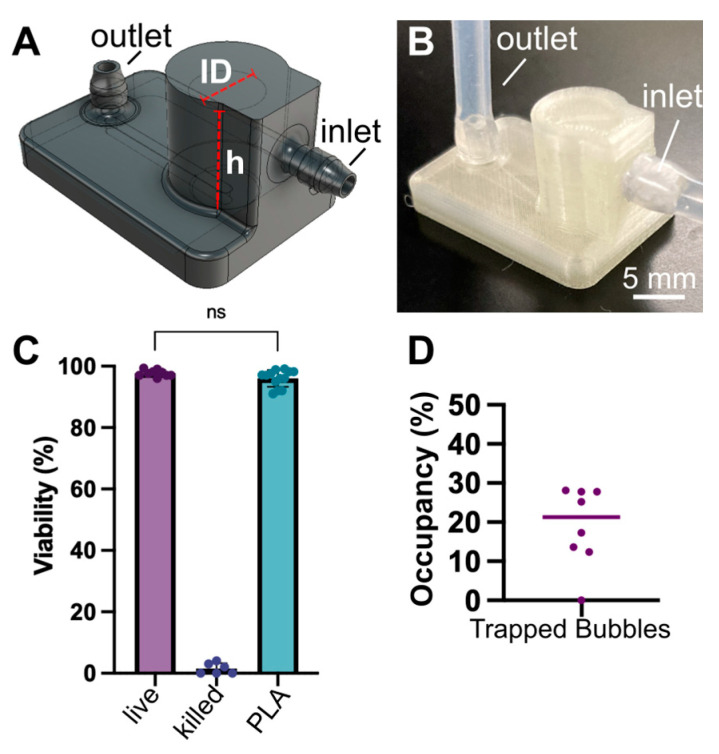
3D printed degassers provided prolonged bubble removal and were cytocompatible. (**A**) Design schematic of degasser intended for FDM fabrication. ID = 5 mm, h = 7 mm, barbs for 1/16 in ID tubing at inlet and outlet. (**B**) Photo of FDM printed bubble trap with tubing connected. (**C**) Quantification of viability of human T cells cultured in conditioned media that was collected as effluent from degassers, compared to live and ethanol-killed plate controls. Viability was defined as percent of cells positive for calcein. One-way ANOVA, ns (*p* = 0.1883). (**D**) The percent of the degasser chamber occupied by bubbles after 14 days of media perfusion at 37 °C. All data points are at less than 30% of total available capacity, with a median of 21.3% capacity. Each data point represents one bubble trap, and the line represents the median.

For fabrication at low to moderate throughput, we turned to 3D printing. 3D printing is a relatively rapid and inexpensive approach for fabrication of microfluidic modules, provided they are of sufficiently large dimensions to be compatible with the resolution of the printer [20,21]. Here we used a common and affordable FDM 3D printer (Creality, Ender 3 v2 Neo) and generic PLA filament capable of printing millimeter scale features to produce the degassers (Figure 3A,B) as needed in-house. This proved to be a straightforward approach for fabrication, requiring about 1 h per individual degasser when using a 0.2 mm nozzle head. We typically printed up to 10 degassers in sequence in a single build. Theoretically, with the build size of the Ender 3 FDM printer, the print capacity could be increased to include >100 traps for a single build, and settings could be optimized for speed as needed.

We found that the rate of success for printing leak-free devices with accurate geometries was dependent on the print settings and PLA type (settings are detailed Section 2.3). Some optimization of printer settings would likely be required for different printers and environments, e.g., to ensure good bonding between layers to provide a watertight material. Nevertheless, it is expected that prints would be fairly consistent across platforms that allow for extrusion of PLA with nozzle sizes between 0.1 and 0.4 mm.

To ensure leak-free tubing connections, several inlet designs were tested for the FDM printed degasser, including a simple hole port, a raised port design [22], integration of silicone adaptors (Male mini luer tube tuck connector, LabSmith, Livermore, CA, USA), and printed barbs. Insertion of tubing into a printed inlet port (receptacle) proved ineffective, as the stiff receptacle did not conform to tubing. Since PLA is a rather rigid plastic, we found the best solution was to design a barb connection that allowed elastic tubing to stretch snuggly over the connection (Figure 3A,B). PTFE tape was used to further smooth the surface of the PLA barb and increase the conformal contact between the tubing and barb, as is common for other plumbing connections.

The 3D printed degassers were tested for biocompatibility and efficacy of bubble removal in the same manner as the machined degassers. Similar to the machined degassers, PLA degassers were cytocompatible when compared to live controls (Figure 3C and Figure S1), and the devices were able to effectively trap bubbles over the course of two weeks with less than 30% occupancy of the chamber (Figure 3D). Assuming again a constant rate of bubble formation under the same conditions, it would take upwards of 5 weeks to fill the capacity of the chamber to 50%. This time could likely be extended further by further equilibrating the media prior to use, thus reducing the number of bubbles needing to be trapped within the chamber.

We also tested the feasibility of using resin 3D printing to manufacture the bubble traps (Figure S2), and found that once again, the inlet connections and overall design were successful in removing bubbles. However, the photocrosslinked materials were found to release cytotoxic components into the effluent and negatively impacted viability unless extensively post-treated (Figure S3), consistent with prior reports with T cells [21,23]. Though recent findings indicate that the application of parylene-C to resin 3D printed devices may mitigate the toxicity issue [24], we chose to proceed with FDM printing as a more widely accessible and less costly option.

Overall, the FDM 3D printed bubble traps were effective in bubble removal, biocompatible, and could be adapted to accommodate commercial tubing by printing a barb. FDM 3D printing is more widely available than machining for small batches, with the primary limitations being occasional inconsistencies in prints (e.g., stringing or print seam disconnection), which could require re-optimization of print settings, and lower optical transparency than polystyrene. On the other hand, additional benefits of 3D printing also included easy adaptability for modifications to adjust size, port type, or chamber ceiling. By printing an open top, for example, it may be possible to introduce a gas permeable membrane atop the chamber for venting, to provide even longer-term degassing without limitations due to trapping capacity.

### 3.3. Proof of Concept Application and Fabrication Comparison

**Figure 4:**
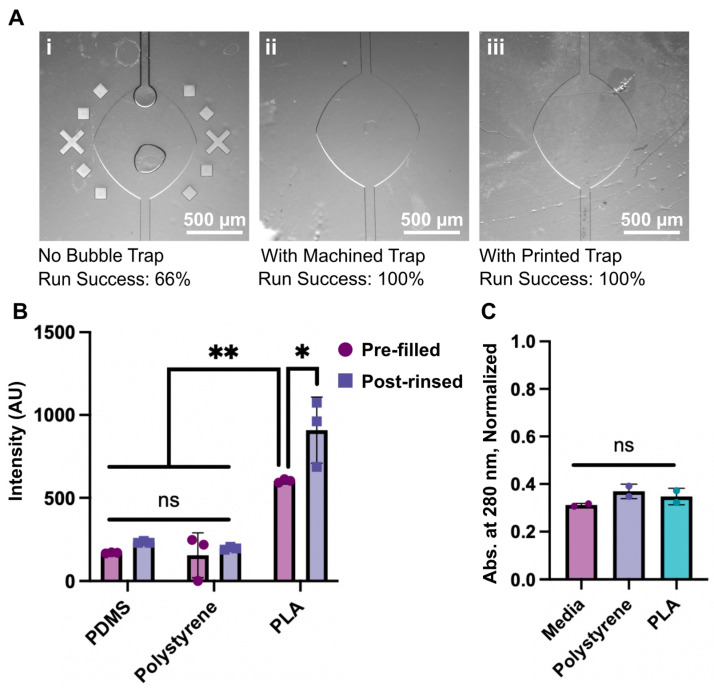
Degassers mitigated bubbles from upstream flow through microfluidic chips, with minimal absorption or leaching. (**A**) Photos of chips after media was perfused overnight (1 µm/min, 20 h, 37 °C) with or without in-line bubble traps (n = 3 chips), with the fraction of chips that contained no bubbles (“run success”) noted below. The X’s and squares in the left image are alignment markers that were included in some of the chips. (**B**) Plot of fluorescent signal from absorption of rhodamine into bubble trap materials before and after 24-h exposure. Two-way ANOVA, ** *p* < 0.0093, * *p* = 0.0377, ns > 0.9998, n = 3. (**C**) UV-Vis absorbance (A_280_) of AIM-V media after overnight perfusion through the traps, compared to non-conditioned media. One-way ANOVA, ns > 0.2297. Points indicate individual samples, bars show the mean and standard deviation.

Having established basic functionality at trapping bubbles and biocompatibility, we put the machined and printed degassers through a series of further tests. First, we tested the degassers for in-line bubble removal. Degassers were placed ahead of a microfluidic chamber that we reported previously for cell culture applications [14]; this chamber includes a lateral broadening that is prone to catching bubbles if any pass through in the media. The media was pre-equilibrated for a shorter time than usual (~2 h rather than 24 h) to provide a rigorous test for bubble removal. After overnight flow of media, a 33% failure rate was observed for chips without degassers, whereas no bubbles were found in the chambers or channels of any chips with degassers (Figure 4A). In these experiments, we observed that all bubbles caught in the degasser remained trapped near the top of its chamber and had a negligible influence on downstream flow rate.

We quantified the potential for the bubble trap materials to absorb molecules from solution, using rhodamine as a test case. To test absorption, bubble traps were filled with a solution of rhodamine dye, allowed to incubate at 37 °C overnight without flow, and rinsed (Figure 4B). A PDMS chip was included for a comparison. Comparing the fluorescent intensity of the materials before and after treatment, PDMS and the machined polystyrene chips experienced little to no absorption of the dye. In contrast, the PLA printed devices did show some uptake of the dye within the walls (Figure 4B and Figure S4), potentially due to small gaps that form between material layers during FDM printing. It is possible that the permeability of the PLA devices could be lessened by increasing the infill percentage or changing the infill geometry within the bubble trap to create a better seal between each printed layer.

As mentioned above, leaching of molecular components into culture media is a challenge for some materials [25,26,27]. To evaluate leaching of fabrication materials into media from the bubble traps, media was collected from the traps after overnight perfusion and evaluated with UV-Vis. All media samples showed similar spectra before and after overnight perfusion, suggesting no additional photoactive materials were leached from either bubble trap (Figure 4C and Figure S5). This result is consistent with the biocompatibility of the materials observed in Section 3.1 and Section 3.2. We note that we cannot exclude the possibility that non-photoactive compounds leached into the media, but leaching is typically not reported as a problem for PLA or polystyrene materials in cell culture.

Finally, pressure tests determined that both varieties of degassers were able to withstand fluid flow that was pumped with an applied pressure of at least 1000 mbar (100 kPa), which was the maximum pressure applied, with <1000 mbar being considered as typical working conditions [28,29]. No leaking or bursting was observed at tubing connections, inlets, or the body of the degasser at this fluid pressure. Flow rates at the maximum applied pressure were estimated to be >1 mL/min. It was observed that at high flow rates (>1 mL/min), bubbles entrained in the fluid were carried towards the bottom of the chamber and were more likely to disrupt the flow path.

Overall, we found both machined and 3D printed degassers were effective at preventing bubbles from entering downstream chips, did not leach detectably into the media, and maintained fluidic integrity under rapid pressure-driven flow. The machined degassers did not absorb the tester molecule, while the FDM printed degassers absorbed a small amount, potentially due to inadequate filling of the interior material during printing. The optimal choice of fabrication method thus primarily depends on the required throughput and material properties (Table 1).

## 4. Conclusions

Adaptation of a passive degassing design based on buoyancy of bubbles enabled simple, reproducible, and scalable fabrication, without the need for integration of pressure controllers, vacuum sources, or valves. Both the machined and 3D printed degassers could be inserted modularly upstream of a microfluidic chip, and were found to be effective for simple, efficient degassing. The modular design is expected to be compatible with any downstream microfluidic device that is perfused by pressure-driven flow from an upstream pump, including for organ-on-chip applications, other microscale cell cultures, and other uses of microfluidics that are compatible with PLA and polystyrene. When built from these cytocompatible materials, the machined and printed traps both preserved the viability of cell cultures, while maintaining material and fluidic integrity, though the printed traps did absorb some of the tracer dye. The best choice for fabrication method depended primarily on access to instrumentation and number of traps required. Machined polystyrene may be preferable when a large number of traps or a high degree of consistency and transparency is required, whereas the 3D printed PLA traps offer a more economical and accessible version for a moderate number of traps. We envision that this work will provide researchers with a simplified bubble removal method that is both affordable and compatible with current flow control and chip setups.

## Figures and Tables

**Table 1:** Comparison of Bubble Trap Fabrication Strategies.

	CNC Machining	FDM 3D Printing	Resin 3D Printing	Soft Lithography
Est. cost (USD, per degasser)	~$1’s (at 1000s scale)–~$100’s (at 10s scale) ^1^	$0.02	$1.25	$2.50
Est. fabrication time (25 chips)	2–3 weeks ^1^	12–25 h ^2^	40 min	3–5 days ^3^
Material	Polystyrene	Polylactic acid	Photocurable acrylate resins	Polydimethyl-siloxane
Material elasticity	Low	Low	Moderate	High
Tubing connections	Inserted adapters, barbed fittings	Integrated barbs	Direct raised ports	Direct ports or fittings
Optical transparency	High	Moderate	Moderate—High	High

^1^ Outsourced, varies by build number. ^2^ Depends on nozzle size. ^3^ Depends on molds available.

## Data Availability

Design files created for this work can be found at: https://dataverse.lib.virginia.edu/dataverse/PompanoLab (accessed on 8 February 2023).

## Supplementary Materials

The following supporting information can be downloaded at: https://www.mdpi.com/article/10.3390/mi14020435/s1, Figure S1: Images for cytocompatibility tests; Figure S2: Schematic of design for resin 3D printed degasser; Figure S3: Viability data for resin 3D printed bubble traps; Figure S4: Images from rhodamine absorption experiment; Figure S5: UV-Vis spectra for conditioned media. Design files are available at the link below in Data Availability Statement.

## References

[1] Pereiro I., Khartchenko A.F., Petrini L., Kaigala G.V. (2019). Nip the Bubble in the Bud: A Guide to Avoid Gas Nucleation in Microfluidics. Lab Chip.

[2] Qi Y., Klausner J.F. (2005). Heterogeneous Nucleation With Artificial Cavities. J. Heat Transf..

[3] Gao Y., Wu M., Lin Y., Xu J. (2020). Trapping and Control of Bubbles in Various Microfluidic Applications. Lab Chip.

[4] Karlsson J.M., Gazin M., Laakso S., Haraldsson T., Malhotra-Kumar S., Mäki M., Goossens H., van der Wijngaart W. (2013). Active Liquid Degassing in Microfluidic Systems. Lab Chip.

[5] Liu C., Thompson J.A., Bau H.H. (2011). A Membrane-Based, High-Efficiency, Microfluidic Debubbler. Lab Chip.

[6] Yang Z., Matsumoto S., Maeda R. (2002). A Prototype of Ultrasonic Micro-Degassing Device for Portable Dialysis System. Sens. Actuators A Phys..

[7] Meng D.D., Kim J., Kim C.-J. (2006). A Degassing Plate with Hydrophobic Bubble Capture and Distributed Venting for Microfluidic Devices. J. Micromech. Microeng..

[8] Skelley A.M., Voldman J. (2008). An Active Bubble Trap and Debubbler for Microfluidic Systems. Lab Chip.

[9] Wu H.-W., Lin X.-Z., Hwang S.-M., Lee G.-B. (2009). The Culture and Differentiation of Amniotic Stem Cells Using a Microfluidic System. Biomed. Microdevices.

[10] Williams M.J., Lee N.K., Mylott J.A., Mazzola N., Ahmed A., Abhyankar V.V. (2019). A Low-Cost, Rapidly Integrated Debubbler (RID) Module for Microfluidic Cell Culture Applications. Micromachines.

[11] Sugiura S., Edahiro J., Kikuchi K., Sumaru K., Kanamori T. (2008). Pressure-Driven Perfusion Culture Microchamber Array for a Parallel Drug Cytotoxicity Assay. Biotechnol. Bioeng..

[12] Zheng W., Wang Z., Zhang W., Jiang X. (2010). A Simple PDMS-Based Microfluidic Channel Design That Removes Bubbles for Long-Term on-Chip Culture of Mammalian Cells. Lab Chip.

[13] Markoski A., Wong I.Y., Borenstein J.T. (2021). 3D Printed Monolithic Device for the Microfluidic Capture, Perfusion, and Analysis of Multicellular Spheroids. Front. Med. Technol..

[14] Ortiz-Cárdenas J.E., Zatorski J.M., Arneja A., Montalbine A.N., Munson J.M., Luckey C.J., Pompano R.R. (2022). Towards Spatially-Organized Organs-on-Chip: Photopatterning Cell-Laden Thiol-Ene and Methacryloyl Hydrogels in a Microfluidic Device. Organs Chip.

[15] Agostini M., Lunardelli F., Gagliardi M., Miranda A., Lamanna L., Luminare A.G., Gambineri F., Lai M., Pistello M., Cecchini M. (2022). Surface-Acoustic-Wave (SAW) Induced Mixing Enhances the Detection of Viruses: Application to Measles Sensing in Whole Human Saliva with a SAW Lab-On-a-Chip. Adv. Funct. Mater..

[16] Kim J., Choi H., Kim C., Jin H., Bae J., Kim G. (2018). Enhancement of Virus Infection Using Dynamic Cell Culture in a Microchannel. Micromachines.

[17] Koch E.V., Ledwig V., Bendas S., Reichl S., Dietzel A. (2022). Tissue Barrier-on-Chip: A Technology for Reproducible Practice in Drug Testing. Pharmaceutics.

[18] Kreß S., Schaller-Ammann R., Feiel J., Priedl J., Kasper C., Egger D. (2020). 3D Printing of Cell Culture Devices: Assessment and Prevention of the Cytotoxicity of Photopolymers for Stereolithography. Materials.

[19] Knight E., Przyborski S. (2015). Advances in 3D Cell Culture Technologies Enabling Tissue-like Structures to Be Created in Vitro. J. Anat..

[20] Ngo T.D., Kashani A., Imbalzano G., Nguyen K.T.Q., Hui D. (2018). Additive Manufacturing (3D Printing): A Review of Materials, Methods, Applications and Challenges. Compos. Part B Eng..

[21] Musgrove H.B., Catterton M.A., Pompano R.R. (2022). Applied Tutorial for the Design and Fabrication of Biomicrofluidic Devices by Resin 3D Printing. Anal. Chim. Acta.

[22] Musgrove H., Pompano R. (2022). Threadless Chip-to-World Connections on Resin 3D Printed Microscale Devices. https://blogs.rsc.org/chipsandtips/2022/11/02/threadless-chip-to-world-connections-on-resin-3d-printed-microscale-devices.

[23] Cook S.R., Musgrove H.B., Throckmorton A.L., Pompano R.R. (2022). Microscale Impeller Pump for Recirculating Flow in Organs-on-Chip and Microreactors. Lab Chip.

[24] O’Grady B.J., Geuy M.D., Kim H., Balotin K.M., Allchin E.R., Florian D.C., Bute N.N., Scott T.E., Lowen G.B., Fricker C.M. (2021). Rapid Prototyping of Cell Culture Microdevices Using Parylene-Coated 3D Prints. Lab Chip.

[25] McDonald G.R., Hudson A.L., Dunn S.M.J., You H., Baker G.B., Whittal R.M., Martin J.W., Jha A., Edmondson D.E., Holt A. (2008). Bioactive Contaminants Leach from Disposable Laboratory Plasticware. Science.

[26] Carter S.-S.D., Atif A.-R., Kadekar S., Lanekoff I., Engqvist H., Varghese O.P., Tenje M., Mestres G. (2020). PDMS Leaching and Its Implications for On-Chip Studies Focusing on Bone Regeneration Applications. Organs Chip.

[27] Venzac B., Deng S., Mahmoud Z., Lenferink A., Costa A., Bray F., Otto C., Rolando C., Le Gac S. (2021). PDMS Curing Inhibition on 3D-Printed Molds: Why? Also, How to Avoid It?. Anal. Chem..

[28] Wang L., Zhang M., Yang M., Zhu W., Wu J., Gong X., Wen W. (2009). Polydimethylsiloxane-Integratable Micropressure Sensor for Microfluidic Chips. Biomicrofluidics.

[29] Hsu M.-C., Mansouri M., Ahamed N.N.N., Larson S.M., Joshi I.M., Ahmed A., Borkholder D.A., Abhyankar V.V. (2022). A Miniaturized 3D Printed Pressure Regulator (ΜPR) for Microfluidic Cell Culture Applications. Sci. Rep..

