# Supplementary material for: A Scalable, Modular Degasser for Passive In-Line Removal of Bubbles from Biomicrofluidic Devices
Source: Micromachines (Basel). 2023 Feb 11;14(2):435. doi: 10.3390/mi14020435 (PMC9964747; doi:10.3390/mi14020435)
Supplement: Supplementary file 1 [file micromachines-14-00435-s001.zip › micromachines-2144439-supplementary.pdf]

Electronic Supplementary Materials for

A scalable, modular degasser for passive in-line removal of bubbles from biomicrofluidic devices

Hannah B. Musgrove,[1] Amirus Saleheen,[1] Jonathan M. Zatorski,[1] Abhinav Arneja,[2] Chance John Luckey,[2] and Rebecca R. Pompano[1,*]

University of Virginia Charlottesville, VA 22904, USA

(1) Department of Chemistry
University of Virginia
PO BOX 400319
Charlottesville, VA 22904

(2) Department of Pathology
University of Virginia School of Medicine

Corresponding author: e-mail: rpompano@virginia.edu, Tel: 434-982-1825

Contents

- Bubble trap designs, hyperlink to data files
- Supplemental Figure S1: Images from cytocompatibility tests
- Supplemental Figure S2: Schematic of design for resin 3D printed degasser
- Supplemental Figure S3: Viability data for resin 3D printed bubble traps
- Supplemental Figure S4: Images from rhodamine absorption experiment
- Supplemental Figure S5: UV-Vis spectra for conditioned media

Bubble Trap Designs

Design files for both FDM and DLP 3D printed degassers are available for download on our database site at the following URL:
https://dataverse.lib.virginia.edu/dataverse/PompanoLab

Supplemental Figures

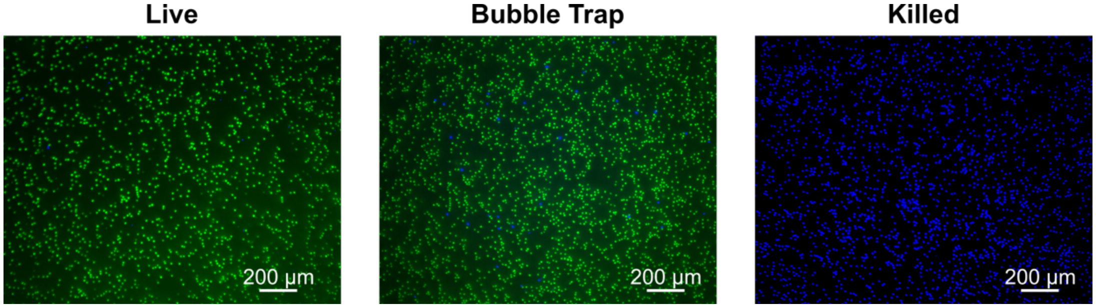

Figure S1 Representative images from microscopic assessment of cell viability. Cells were cultured for 24 hr in fresh media or effluent from a bubble trap, after which they were stained with Calcein (green) and DAPI (blue) to identify live and killed cells, respectively. (*left)* Image from cell culture in fresh media. *(center)* Image from cell cultured in media conditioned by overnight perfusion through an FDM printed bubble trap. Images from machined traps looked similar (not shown). (*right)* Image of ethanol-treated cells used as a negative control.

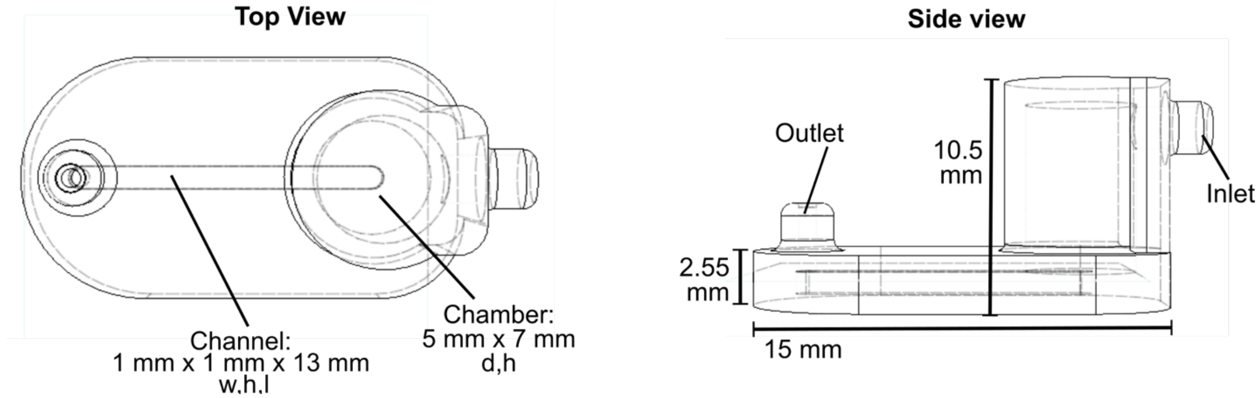

Figure S2 Schematic of design for fabrication of the bubble trap by digital light processing (DLP) 3D printing.

Figure S3 Biocompatibility testing of resin printed bubble traps. Bubble traps were printed with a CADWorks3D MiiCraft Ultra 50 Series digital light processing 3D printer, using FormLabs Dental Resin. Prints were washed in isopropyl alcohol and UV post-cured with the FormWash and FormCure, according to FormLab's suggested protocol. Prints were used without further treatment (non-treated), or were soaked for 72 hours in PBS at 37°C to leach toxins out of the materials prior to use. Media was flowed through the bubble traps, and effluent was collected as described in the main text and used to culture human T cells for 24 hr. Viability was defined as percent of cells positive for calcein versus total cells. Live and ethanol-killed plate controls were compared to non-treated and PBS soaked bubble traps. One-Way ANOVA, ns p >0.0916.

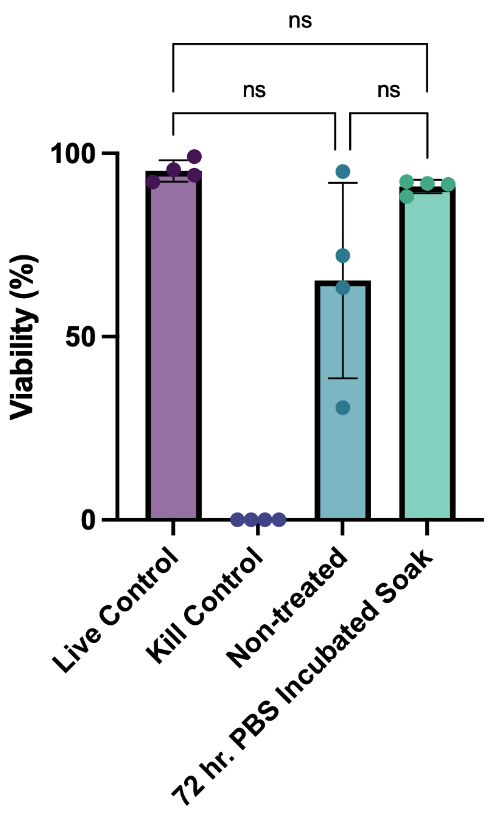

We conclude that post-treatment of resin printed bubble traps is required if used upstream of cell culture devices, and further note that the extent of leaching required likely depends on the volume and geometry of the print, as well as the cell type of interest.

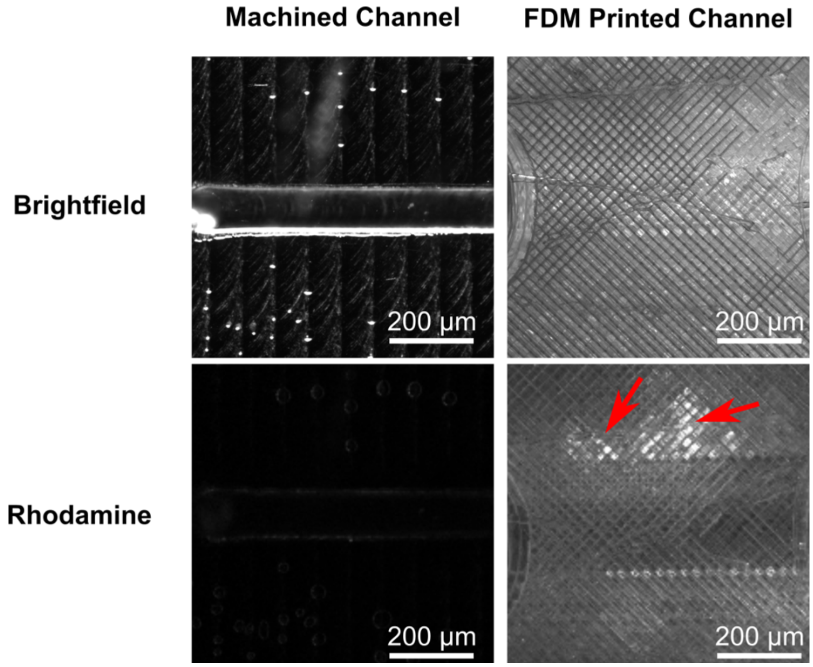

Figure S4 Representative images from rhodamine absorption experiment shown in **Figure 4B** in the main text. Brightfield and fluorescent channels are shown separately. Red arrows are used to highlight areas of visible rhodamine dye absorption near the channel of 3D printed degasser. We speculate that using tighter and denser infills may lessen the permeability of the printed material if this poses a problem.

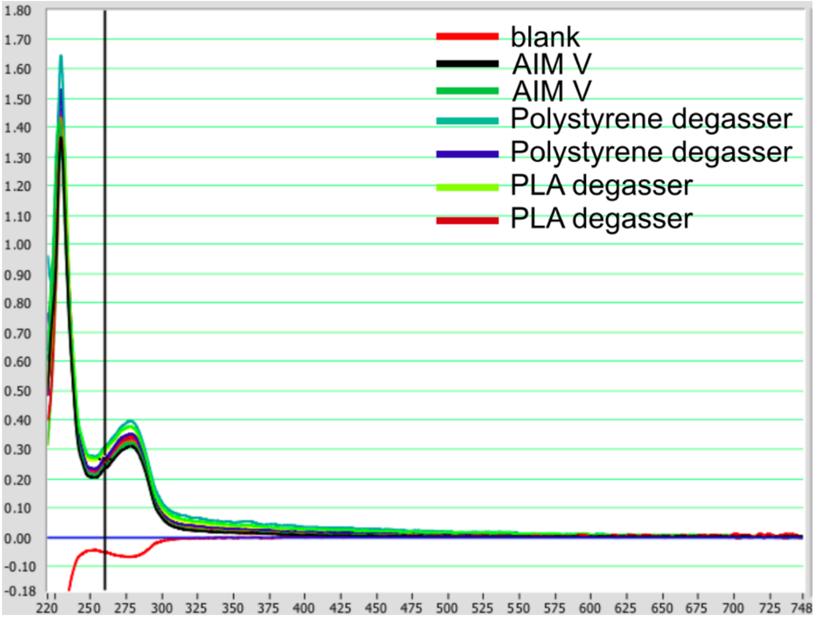

Figure S5 UV-vis spectra corresponding to the experiment shown in **Figure 4D** in the main text. Range is from 200 nm to 700 nm. Cursor (black, vertical line) is at default position of 260 nm.
